# The Multi-Component Structure of Core Strength

**DOI:** 10.3390/jfmk9040249

**Published:** 2024-11-28

**Authors:** Sarah Schulte, Jessica Bopp, Volker Zschorlich, Dirk Büsch

**Affiliations:** 1 Institute of Sport Science, School of Humanities and Social Sciences, Carl von Ossietzky Universität Oldenburg, 26129 Oldenburg, Germany; jessica.bopp@uni-oldenburg.de (J.B.); or volker.zschorlich@uni-rostock.de (V.Z.); dirk.buesch@uni-oldenburg.de (D.B.); 2 Institute of Sport Science, Universität Rostock, 18057 Rostock, Germany

**Keywords:** principal component analysis, diagnostics, force-differentiated approach, core power, core endurance, maximal core strength

## Abstract

Background/Objectives: Core strength diagnostics often focus on measuring core endurance rather than maximal core strength or core power. This study investigates whether core strength can be considered as a general ability that can be measured by a single core strength test or whether it needs to be differentiated into several components. Methods: Forty-two adult sports students (*n*_female_ = 20; *n*_male_ = 22; age: 24.0 ± 2.9 years; body height: 179.0 ± 9.8 cm; body mass: 75.2 ± 12.7 kg; body fat: 18.0 ± 6.8%) participated in two randomized testing sessions in a laboratory setting. Standard measurements, such as peak rate of force development (pRFD), maximal voluntary contraction (MVC), and holding time, were taken isometrically during four exercises (ventral, dorsal, and lateral right and left). Results: A principal component analysis (PCA) extracted three principal components from twelve different core strength variables. The three identified components explained 73.3% of the total variance and were labeled as (a) maximal core strength, (b) core endurance, and (c) core power. Conclusions: The results suggest three principal components of the core strength construct, as well as their differentiation, may be imperative. These findings should be taken into account in sport science and sports practice as they may be helpful in planning sport-specific diagnostic, performance-oriented training, and injury prevention programs.

## 1. Introduction

The core is described as a muscular box, corset, or cylinder, with the abdominal muscles in the front, the paraspinal and gluteal muscles in the back, the pelvic floor and the hip at the bottom, and the diaphragm at the top [1,2,3,4,5]. Panjabi [6] classified the core functionally into three independent subsystems, namely the active neural, passive, and active muscular subsystems, which provide spinal stability during movement or as a reaction to internal and external perturbations. Consequently, the active neural subsystem regulates the activation of the core muscles via feed-forward and feedback mechanisms. The passive subsystem comprises the vertebral ligaments, intervertebral disks, and facet joints between adjacent vertebrae, which provide passive stiffness for lumbar spine stability. The active muscle system provides active lumbar spine stiffness through the core’s musculature, thereby supporting the passive subsystem [2,6,7,8].

The active muscle system of the core can be further differentiated into a global and local system as well as an axial–appendicular transfer system [3,7,8]. The primary function of the local system is to coordinate and control segmental motion, thereby ensuring intersegmental stability. The global system provides multisegmental stability, enabling a greater range of motion during movement and producing larger forces to stimulate movement during dynamic activities [3,7,9,10]. The axial–appendicular muscles connect the core via the pelvic girdle and shoulder girdles to the lower and upper extremities and are responsible for transferring the torques and angular momentum from single-joint and multi-joint muscles to the distal segments of the body during sports performance [7,8]. The global, local, and axial–appendicular transfer systems work synergistically to achieve proximal stability in the core for distal mobility and power to the upper and lower extremities [11]. Thus, the core can be regarded as a link and the center of almost all kinetic chains, playing an important role in maximizing upper and lower extremity functions and sports performance [7,11,12,13,14].

Upon an examination of core anatomy and the functions of the core muscles, it becomes evident that maintaining core stability requires core strength, which is controlled by the central nervous system through neuromuscular control. Core strength is widely regarded as an important component of sports performance and injury prevention or rehabilitation [14,15,16,17]. Core strength is related to core muscle mass and contractile potential, which produces forces and leads to stable and secure positions of the spinal column, thereby providing core stability [2,7,18]. On the basis of the classification of strength as a general motor ability [19], it can be assumed that core strength may include various components, such as maximal core strength, core endurance, and core power [16,20,21,22]. With reference to the general definition of the term ‘strength’ of Siff [23], core strength can be defined as the ability of a core muscle or a core muscle group to generate muscular force, whereby maximal core strength means the ability of a particular core muscle group to produce a maximal voluntary muscle action in response to optimized motivation against an external load. Core power refers to the rate at which work is performed and can be considered as force times velocity [24], whereas core endurance can be defined as the capacity to perform repeated muscle actions with a given load or exerting force for an extended time period [24].

The required expression of core strength, including maximal core strength, core endurance, and core power, probably depends on the demands within a specific sport or task [16]. For example, in sports practice, ball sports players need to produce the highest power at higher velocities and low loads, combat sports athletes need to generate a high force in a short period of time, and grappling sports athletes need to produce the highest power at higher loads [16]. On the basis of sensory information, muscle activation is executed with precision, occurring at the appropriate time, for the correct duration, and with the appropriate combination of forces to control the position or movement of the body in response to internal and/or external perturbations, as well as expected and unexpected perturbations. Accordingly, precise neuromuscular control of the production and transfer of core strength allows core stability in integrated kinetic chain activities [2,13,18,22,25].

However, to the authors’ knowledge, it has not yet been systematically examined whether core strength can be regarded as a general ability or whether a classification of core strength in individual components appears necessary. The current research includes a lack of standardized measurement systems for assessing core strength [5,26,27]. The choice of test for measuring core strength is often an arbitrary selection [28]. The majority of studies have employed core endurance tests rather than maximal core strength and core power tests to quantify core strength [5,14,16,28,29,30]. As a result of this, the current literature contains limited and in parts conflicting scientific evidence regarding the efficacy of core strengthening and stabilization exercises in enhancing athletic performance or preventing injuries [14,16,29,31,32,33]. Thus, a classification of core strength can only be supposed. Wirth et al. [18] called for greater attention to be paid to the muscular strength components of the core and their associations in the field of sports.

A systematic measurement of core strength is therefore indicated to identify the components of core strength. The measurement of various variables of core strength and the delineation of their interrelationships may facilitate the development of core strength diagnostics or the selection of core strength tests for practitioners (e.g., coaches and physical therapists) [16]. Further, it can be assumed that the specific requirements of a population (e.g., age, sex, and sport) may change the magnitude and nature of the potential relationship between core strength variables [34]. Studies that investigate the differences in the maximal isometric strength, endurance, and power of core muscles in relation to age, sex, and sports are scarce [16,26,35,36,37,38]. Furthermore, these studies measure maximal core strength or core endurance instead of choosing a more complex core strength measurement. A larger data base concerning these aspects would permit the design of more effective performance-oriented training, injury prevention, and rehabilitation programs that are tailored more closely to individual and sport-specific needs [36].

A limited number of studies have examined the relationships between core strength components. In a study by Saeterbakken et al. [21], the Biering–Sørensen test, abdominal flexion test, and lateral flexion test were used to evaluate core endurance and maximal isometric core strength [39,40]. The researchers reported no correlation between core strength components and concluded that these components should be trained separately given to the demands of the sport-specific task [21]. Similarly, Roth et al. [35] investigated the relationship between the maximal core strength and core endurance in a sample of 39 German high-level footballers. The researchers employed the Bourban test [41] to assess core endurance through prone (ventral), side (lateral), and back extension exercises (dorsal) in a lying position. The maximal core strength was quantified with four distinct testing machines, with the objective of determining the maximal force in trunk extension (dorsal) and flexion (ventral), rotation to the left and right sides, and left and right lateral flexion exercises in a seated position. The results of the maximal core strength and core endurance tests did not demonstrate any correlation. The authors noted that the strength of the extremities contributed to performance in the prone and side plank exercises, as they influence the duration of performance [35].

Tomčić et al. [42] employed a battery of tests to assess the MVC force for the back, anterior abdominal, and lateral abdominal muscles in standing and seated positions via isometric dynamometry; the explosive power of the back, anterior abdominal, and lateral abdominal muscles in standing and seated positions via static and dynamic medicine ball throws in four directions (forward, backward, right, and left); and the holding time in a static core muscle endurance test battery for back, anterior abdominal, and lateral abdominal muscles in a lying position via the procedure of McGill et al. [37]. Tomčić et al. [42] used principal component analysis to identify core power, core endurance, and maximal core strength in a seated position, as well as maximal core strength in a standing position as principal components of twenty variables. The correlations between the maximal core strength in seated and standing positions were low and negative, indicating that these two components are independent. Furthermore, all four identified components showed no statistically significant correlations in their principal component analysis.

With respect to the cited studies, there were methodological differences in determining the holding time, power, and MVC of the core muscles. It could be assumed that different testing procedures and test positions (seated, standing, and lying) do not address the same core muscles with similar intensities. To recruit the same core muscle groups for measuring holding time, MVC, and power, the same test positions (seated, standing, or lying position) and test exercises (ventral, dorsal, and lateral) would be necessary. The studies of Tomčić et al. [42], Bucke et al. [43], and Saeterbakken et al. [21] reported the efficacy of standardized test positions for assessing holding time and MVC via isometric testing. The tests employed (Biering–Sørensen test, abdominal flexion test, and lateral flexion test) were frequently utilized in previous studies and have become reliable instruments for measuring maximal core strength and core endurance [21,40,42,43]. Furthermore, the use of the lateral flexion test for measuring the core endurance of abdominal oblique muscles appears to be more appropriate because the strength or stability of the shoulder is more often the limiting factor than the muscular endurance of the abdominal oblique muscles when the Bourban test [41] and/or the McGill test [37] are performed in side bridge exercises [35].

To address the issues described in relation to the lack of an established standardized measurement for the assessment of core strength components and the limited evidence regarding the relationships between these components, our study aims to measure the holding time, maximal voluntary contraction (MVC), and peak rate of force development (pRFD) for different core muscle groups in a test battery. All core strength parameters were measured at the same test position within the test battery. This approach enables the determination of the components that constitute the core strength construct. To the best of our knowledge, no previous study has evaluated the associations among holding time, MVC, and pRFD variables in healthy male and female sports athletes. On the basis of previous studies [21,35,42], we hypothesized that the aforementioned variables (1) can be grouped into different components that predominantly constitute the construct of core strength and (2) show no relationships with each other.

## 2. Materials and Methods

### 2.1. Participants

A size of *N* = 40 was determined a priori using the formula of Giraudeau and Mary [44] (Supplementary Materials, sample size calculation). Taking into account potential dropouts from the study, forty-four adult sports students from the University of Oldenburg were included. Subjects were recruited through public notices and personal enquiries. Participants were included in the study if they were sports students who were regularly physically active and had no injuries in the core area or lower and upper extremities within the past year and at the time of measurements. Furthermore, participants were allowed to take part in the experiment if they had no acute or chronic back pain. Two subjects were excluded from further analysis because of implausible extreme values for technical reasons. The final analysis was conducted from the data of forty-two participants (*n*_female_ = 20; *n*_male_ = 22; age: 24.0 ± 2.9 years; body height: 179.0 ± 9.8 cm; body mass: 75.2 ± 12.7 kg; body fat: 18.0 ± 6.8%). The participants were active in various sports. Fifteen of the forty-two subjects dedicated 49.7 ± 51.1 min per week to specifically training their core strength. This study was conducted in accordance with the Declaration of Helsinki, and the Research Ethics Committee of the Carl von Ossietzky University of Oldenburg approved the protocol (approval number: Drs.EK/2020/035-08, 21 April 2023).

### 2.2. Procedures

All the subjects took part in two 60 min sessions in a laboratory of the Department of Training and Exercise Science of the University of Oldenburg. Prior to the commencement of the experiment, the subjects were informed about the procedures and provided written informed consent for the following experiment. On the first day of testing, the subjects completed a questionnaire including personal data, sporting background, and injury history. Subsequently, anthropometric measurements (body mass, body height, and body composition) were conducted via the Inbody270 (Inbody Co., Ltd., Seoul, Republic of Korea) and a stadiometer (Seca 217, Seca GmbH & Co. KG, Hamburg, Germany). Prior to core strength measurement, in both testing sessions, the subjects completed a supervised five-minute warm-up program to activate the whole musculature of the body, especially the core. One of the two testing sessions included the measurement of holding time, while the other contained the MVC and the pRFD measurements. The order of the testing sessions was randomized. There were at least seven days between the two testing sessions. The MVC, pRFD, and holding time were measured isometrically through flexion (abdominal muscles), extension (back muscles), and lateral flexion exercises (oblique muscles) [21,40]. Throughout the test executions, the participants were verbally encouraged by the examiner to hold the position for as long as possible during the holding time measurement and to pull on the force sensor as fast and strongly as possible during the pRFD and MVC measurements. Two examiners familiar with the test procedure conducted the experiment.

#### 2.2.1. Holding Time Measurement

**Figure 1:**
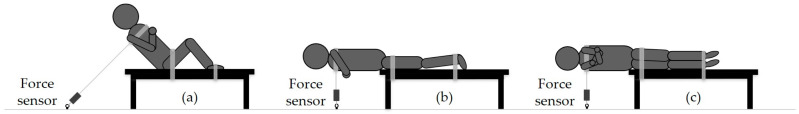
Isometric measurement of holding time, maximal voluntary contraction (MVC), and peak rate of force development (pRFD) in (**a**) flexion, (**b**) extension, and (**c**) lateral flexion positions.

The Biering–Sørensen test was employed to assess the endurance of the back extensor muscles, whereas the abdominal flexion test was used to evaluate the endurance of the abdominal flexor muscles. The lateral flexion test was utilized to determine the endurance of the oblique muscles on both the right and left sides [21,40,42]. The participants were instructed to maintain the different test positions as long as possible (see Figure 1). If the subject was unable to maintain the correct position the first time, the examiner issued a warning. If the subject failed to maintain the position a second time, the test was terminated. To ensure standardized test positions, a manual goniometer was employed. The holding time was recorded in seconds via a hand stopwatch. Following test termination, the body area of exhaustion was requested by the examiner. The order of test positions was identical for each participant and corresponded to the following program: core abdominal flexion, core back extension, lateral flexion right, and lateral flexion left. Each of the aforementioned tests were performed once, owing to their moderate to high reliability (*ICC* = 0.73–0.91) [21]. Before the test trial in each position, the participants were permitted practice trials to familiarize themselves with the test procedure. In the practice trials, the test positions were held for a maximum of only five seconds to avoid fatigue effects. Between each test position, the participants rested for five minutes [42].

In the flexion position, the participants were seated on a therapy table. Their knees and hips were flexed at 90°, and their trunk was held at an angle of 45° to the therapy table. Their arms were crossed over their chest. Their feet were positioned entirely on the table. The examiner fixed the subjects with two resistance straps at their hip and forefoot. The test started when the subjects displayed the correct posture (see Figure 1a).

In the extension position, the participants laid in a prone position on the therapy table, with their upper body extending beyond the therapy table. Their iliac crest remained stationary on the table, while their arms were crossed over their chest. Their upper body was aligned with their lower body. During test execution, the subjects had no support of their upper body or their arms. The shanks and thighs of the subjects were fixed with two resistance straps by the examiner. The height of the therapy table was adjusted to 50 cm, allowing the subjects to support themselves with their hands on the floor and lean on a box with their upper body, which was removed by the examiner before the test started. The test commenced once the subjects were moved to the correct position (see Figure 1b).

In the lateral flexion position, participants lay laterally on the therapy table, with their lower extremities and hips resting on the table. Their arms were crossed over their chest. Their upper body was held in line with their lower body. The participants had no support of their upper body or arms during the test execution. The examiner secured the subjects at their shanks and thighs with two resistance straps. Owing to the low height of the therapy table, the subjects had the opportunity to support themselves once more before the test began. The test was initiated when the participants were in the correct position (see Figure 1c).

#### 2.2.2. Maximal Isometric Voluntary Contraction and Peak Rate of Force Development Measurement

The MVC and pRFD were measured in the same positions and order as described for the holding time measurement (flexion, extension, and lateral flexion right and left). Consequently, a force sensor (Ergotest Innovation AS, Stathelle, Norway) was attached to the ground and connected via a resistance strap to the trunk of the subject. The strap was positioned directly beneath the subject’s armpits. In the flexion position, the angle between the strap connecting the force sensor and the trunk was between 41 and 45 degrees (see Figure 1a). In the extension and lateral flexion positions, the strap connecting the force sensor and the trunk had an angle of 90° to the ground (see Figure 1b,c).

The angles in the test positions were verified via a goniometer to guarantee the highest possible standardization of the test conditions. The examiner started a countdown, which lasted for three seconds. At the end of the countdown, the participants were instructed to pull from a light preload as hard and fast as possible on the force sensor for a duration of five seconds [45,46] in each of the four test positions (flexion, extension, and lateral flexion right and left). For each test position, participants were required to complete three practice trials with submaximal effort followed by three test trials with maximal effort. A one-minute interval of rest was allowed between each test trial in a single test position. A two-minute interval of rest was taken during the transition between positions.

**Figure 2:**
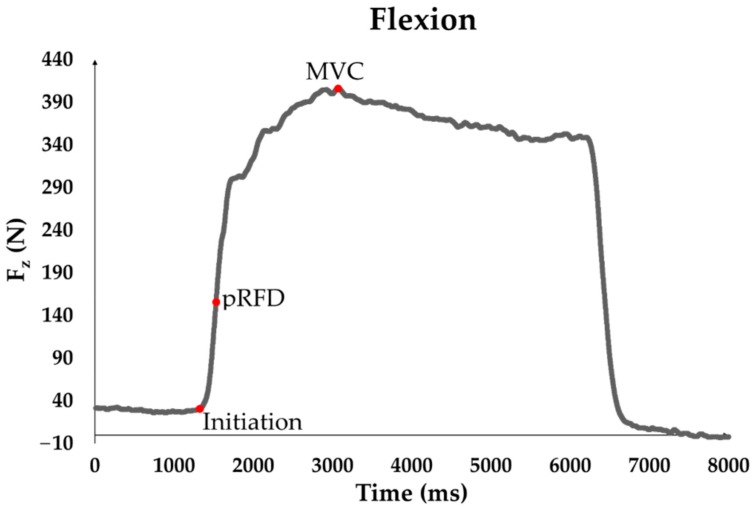
Force–time curve of isometric measurement with relevant parameters (MVC, pRFD) highlighted.

The force curves were processed via MuscleLab software (Version 10.200.90.5097, Ergotest Innovation AS, Stathelle, Norway) with a sampling rate of 1 kHz [46]. After the raw data were exported to Excel, the maximal voluntary isometric contraction was determined as a 20-millisecond moving average in the force–time curve for each test trial. The mean value of the three test trials in each position was used for further analysis. Additionally, the highest rate of force development was determined in force–time curves by a 20-millisecond moving average (see Figure 2) [47,48]. The mean value of the three test trials in each position was utilized for subsequent analysis.

### 2.3. Statistical Analysis

The data were subjected to statistical analysis using JASP [0.18.3., University of Amsterdam, Netherlands]. The data were checked for normal distribution through the Shapiro–Wilk test and examined for skewness, kurtosis, and unimodal distribution [49]. To avoid possible bias caused by the low consistency of the MVC and pRFD variables on the principal component analysis, the relative (e.g., intraclass correlation coefficient) and absolute reliability (e.g., standard error of measurement, coefficient of variation) of the MVC and pRFD measurements were calculated [50,51]. The within-session reliability of the measurements of MVC and pRFD across the three test trials was determined by calculating the intraclass correlation coefficient (*ICC*; calculation model designation by McGraw and Wong [52]: *ICC* (A, k)). The *ICC* values were interpreted according to Koo and Li [53] as poor (<0.50), moderate (0.50–0.75), good (0.75–0.9), or excellent (>0.90). Furthermore, the coefficient of variation (*CV*) was calculated as $\mathrm{CV}=\frac{\mathrm{SD}}{M}\times 100$ and the standard error of measurement (*SEM*) was computed as $\mathrm{SEM}=\mathrm{SD}\times \sqrt{1-\mathrm{ICC}}$ [54] in order to assess the degree of variation between the repeated measures.

A principal component analysis (PCA) was conducted to extract independent components from the intercorrelation matrix of the holding time, MVC, and pRFD variables. A PCA aims to reduce the dimensionality of a dataset, while variables (e.g., force–time variables) of the original dataset that correlate highly with each other are summarized into components. The new linear functions in the form of components maximize the variance and are uncorrelated with each other to preserve as much variability as possible [55,56,57]. Variables which obtained a moderate level of reliability (*ICC* > 0.60) were included in the PCA [44]. First, the Kaiser–Meyer–Olkin test (KMO) and Bartlett’s test of sphericity were employed to assess the sampling adequacy and substantial correlations between variables, justifying the execution of the PCA. An outcome close to the value of 1 indicates that the PCA is a reliable calculation tool for identifying components with high variability [58]. According to the guidelines of Kaiser and Rice [59], KMO coefficients were interpreted as follows: ≥0.9 = marvelous; ≥0.8 = meritorious; ≥0.7 = middling; ≥0.6 = mediocre; ≥0.5 = miserable; and <0.5 = unacceptable. Furthermore, the component extraction methods of the scree plot [60] and Kaiser’s criteria [61] were applied to identify components with high eigenvalues. Orthogonally varimax rotation was used to enhance the structure of the component pattern matrix. For the purpose of facilitating the interpretation of the extracted components, the variables were assigned to the component on which they loaded higher than on the other components [55,57,58]. In addition, a cut-off correlation (*r*-value) of 0.4 was chosen to filter and order variables in the rotated component matrix for those exhibiting more relevant and higher loadings on extracted components [58,62]. Furthermore, the *t*-test for independent samples was employed to ascertain the existence of differences in the holding time, MVC, and pRFD variables between men and women. The level of significance was set at α = 5% because of two-tailed testing (see Supplementary Materials, Table S2 for comparison core strength variables by sex). The interpretation of the effect was based on Hedges’s *g* ranges of 0.10–0.29 = small, 0.30–0.49 medium, and ≥0.5 = large [63].

## 3. Results

Descriptive statistics of the holding time, MVC, and pRFD measurements are shown in Table 1. All data represented were normally distributed by a given skewness < 2 and kurtosis < 7 according to West et al. [49].

The three MVC measurements showed excellent within-session reliability (*ICC* = 0.96–0.99), whereas the *ICC*s for the pRFD measurements were considered good (*ICC* = 0.83–0.88). For the MVC and pRFD variables, *CV* ranges of 5.51–11.67% and 23.94–29.24% were observed, respectively. The *SEM*s for the MVC and pRFD variables were determined as 10.71–14.20 N and 201.36–408.40 N/s, respectively. All the parameters of within-session reliability are presented in Table 2.

**Figure 3:**
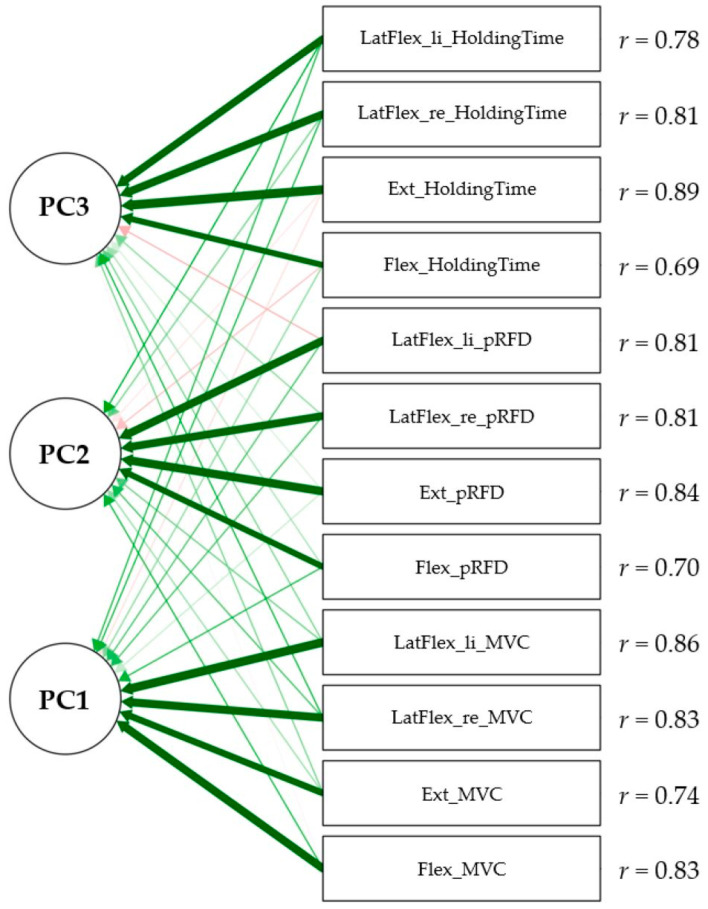
Path diagram of principal component analysis with loadings (*r*) of maximal isometric voluntary contraction (MVC), peak rate of force development (pRFD), and holding time variables on three principal components (PC1, PC2, PC3). Note: Light red colored arrows indicate low negative loadings, light green colored arrows indicate low positive loadings, and dark green wide arrows indicate high positive loadings.

The PCA extracted three independent principal components from the intercorrelation matrix (Supplementary Materials, Table S1). The KMO measure of sampling adequacy verified the analysis, showing a middling KMO coefficient of 0.69 according to Kaiser and Rice [59]. The significant Bartlett’s test of sphericity (*p* < 0.001) indicated that the correlations between the variable of holding time and the MVC and pRFD measurements were substantial for running a PCA. The procedures of Kaiser’s criterion and the scree plot extracted three principal components, which explained 73.3% of the variance of all variables related to the core strength construct. Specifically, the first component (PC1) clarified 44.1%, the second component (PC2) 18.5%, and the third component (PC3) 10.7% of the reported variance. Figure 3 illustrates the three extracted principal components and the loadings of variables on these components in the form of arrows after the component pattern matrix is rotated orthogonally. The greater the distinction in the color and intensity of the arrows, the higher the loading of the individual variable on the respective component. The highest loadings of the variables on the components are listed next to the variables in the displayed path diagram. A review of the path diagram reveals that the MVC variables exhibited the highest loadings (*r* = 0.74–0.86) on PC1, the pRFD variables had the highest loadings on PC2 (*r* = 0.70–0.84), and the holding time variables loaded highest on PC3 (*r* = 0.69–0.89).

Furthermore, statistically significant differences were found in the absolute values of the MVC and pRFD variables between men and women in flexion, extension, lateral flexion right, and lateral flexion left, with higher maximal isometric voluntary contraction forces and pRFDs in men than in women. The results are presented in Table S2 of the Supplementary Materials.

When the holding time in the flexion position was measured, the anterior abdominal muscles (42.9%) were predominantly mentioned as the body area of exhaustion. For the extension position, the lower back muscles (73.8%) were reported as the primary body area of exhaustion. The right (76.2%) and left (83.3%) lateral abdominal muscles were mostly referred to as body areas of exhaustion by the subjects in the right and left lateral flexion positions, respectively.

## 4. Discussion

This study aims to determine whether core strength can be classified in different components and how they can be identified. Thus, it was hypothesized that core strength can be differentiated into components that predominantly constitute the construct of core strength and show no relationships with each other. Within a force-differentiated approach, the holding time, MVC, and pRFD of different core muscle groups (anterior abdominal muscles, back muscles, and oblique muscles) were measured in the same exercises (flexion, extension, and lateral flexion right and left) and in the same position (lying) to identify the principal components of the core strength construct and their relationships. The results are in accordance with the hypotheses of this study.

The PCA with varimax rotation extracted three independent principal components out of twelve variables, which explained 73.3% of the total variance. The first component (PC1) was primarily loaded high with MVC variables. These findings indicate that the first component can be described as ‘maximal core strength’. Furthermore, the second component (PC2) mainly loaded high with pRFD variables. According to these results, the second component can be perceived as ‘core power’. Finally, the holding time variables had their highest loadings on the third component (PC3), so this one can be interpreted as ‘core endurance’. It can be inferred that the identified components ‘maximal core strength’, ‘core power’, and ‘core endurance’ predominantly constitute the core strength construct because of the given high total variance explained.

This study indicates maximal core strength, core endurance, and core power as principal components of core strength in male and female sports students, with the variables of holding time, MVC, and pRFD being used in the same exercises (flexion, extension, and lateral flexion right and left) and in the same position (lying) for isometric measurement. To date, only one study [42] has evaluated maximal core strength in a standing position, maximal core strength in a seated position, core endurance, and core power as principal components of core strength, measured out of twenty variables in ninety-one young male soccer players. Compared with our study, Tomčić et al. [42] used dynamic and static measurements in various exercise positions to determine MVC variables in both seated and standing positions using an isometric dynamometer, distance variables of static and dynamic medicine ball throws, and holding time variables via the McGill protocol [37].

However, it is possible that different core muscles or other adjacent muscles, such as the hip muscles (e.g., hamstrings, gluteal muscles), are recruited with varying intensities in maximal core strength, core endurance, and core power measurements due to differences in test positions and exercise types. De Los Ríos-Calonge et al. [34] argue that young individuals showed different performances even when the same muscle group was assessed in different positions (i.e., standing vs. sitting) or using different types of muscle action (i.e., isometric vs. dynamic) because physical performance highly depends on many different biomechanical demands (i.e., muscle group type, position, muscle action, duration, speed, recovery time, and resistance). To assess the different neuromuscular mechanisms that may occur in different measurement positions and types of muscle actions, an electromyography (EMG) may be implemented in core strength measurement [21].

Moreover, the strength of the upper extremities is more often the limiting factor than the strength of the abdominal oblique muscles in measuring core endurance in the side plank position [35]. Consequently, our study is methodologically guided by the study of Saeterbakken et al. [21], as they employed the same exercises (flexion, extension, lateral flexion) and position (lying) to determine the maximal core strength and core muscle endurance of the anterior abdominal muscles, back muscles, and abdominal oblique muscles. They conducted a lateral flexion test instead of the side bridge exercise in the McGill protocol [37]. The results of this study show that the abdominal oblique muscles were predominantly mentioned as body areas of exhaustion by the subjects in the left and right lateral flexion tests, as core endurance was measured. Thus, further studies are suggested to favor the lateral flexion test over a side bridge test (e g., Bourban test, McGill protocol) to better control that the abdominal oblique muscles are predominantly stressed. Furthermore, it is important to measure the core strength of both sides of the abdominal oblique muscles, as well as the core strength of the anterior abdominal and the back muscles in different sports (e.g., volleyball, golf, and soccer), as imbalances in core strength between the right and left abdominal oblique muscles and between the anterior abdominal and back muscles are potential risk factors for injury (e.g., back pain, overuse injuries) [64,65].

This study found that the majority of test subjects (*n* = 18, 42.9%) identified the anterior abdominal muscles as the body area of exhaustion in the core endurance measurement in the flexion position. However, several individuals also cited the hip flexors (*n* = 9, 21.4%) and the lower back muscles (*n* = 10, 23.8%) as body areas of exhaustion. This means that not all test subjects’ target muscles (anterior abdominal muscles) were primarily addressed by the selected and standardized test position. Modifications to the methodology, including alterations to the instructions or to the position of the body (e.g., the placement of the feet), have the potential to enhance the precision of measurements of core strength in the flexion position. In addition to the core strength measurements conducted by Saeterbakken et al. [21], the peak rate of force development was measured in the present study. Nevertheless, the results of the current study (total variance explained: 73.3%) are consistent with those of Tomčić et al. [42] (total variance explained: 70.0%) because of the high degree of agreement between the total variance explained by the identified components. Additionally, the scope of validity for the assumption that the core strength construct is primarily composed of maximal core strength, core endurance, and core power was expanded.

The PCA of this study yielded results that were relatively consistent with those of other studies [21,35]. Thus, trivial to small correlations were found between the components of core endurance and maximal core strength, as was observed in the cases of Saeterbakken et al. [21] and Roth et al. [35]. Roth et al. [35] used different positions and test procedures to assess maximal core strength and core endurance. In addition to the study of Saeterbakken et al. [21], the current study measured the power of different core muscles isometrically and thereby pursues a systematic methodology for assessing core strength comprehensively. The results indicate that there are mainly trivial to small correlations between core power and core endurance, as well as between core power and maximal core strength. These findings suggest that the measured components are weakly related to each other, which provides preliminary indications. Consequently, it might be beneficial to measure every single core strength component in diagnostics and train these core strength components separately in sport-specific training or injury prevention programs adjusted to individual needs.

In contrast to other core strength diagnostic studies, the current study investigated the core power of different core muscle groups in addition to maximal core strength and core endurance to assess core strength more complexly. The studies conducted by Shinkle et al. [66], Carvalho et al. [67], Ortega-Becerra et al. [68], and Bauer et al. [69] have demonstrated that core strength plays a significant role in an athlete’s ability to create and transfer forces to their extremities. Given that the core is the center of almost all kinetic chains in sporting movements, it is evident that the measurement of core power is crucial for core strength diagnostics, in addition to maximal core strength and core endurance measurements [16,27]. Furthermore, a more comprehensive understanding of the importance of core power in athletic performance can facilitate the design of targeted training and injury prevention programs [16].

It is challenging to make direct comparisons between the results of this study and those of other studies on sex differences in maximal core strength because of the use of different measurement tools and normalized data. However, the findings of this study appear to be consistent with those of other studies that reported a statistically significant higher maximal isometric core strength in flexion, extension, and lateral flexion on the right and left sides in men than in women [35,36]. In contrast to the findings of McGill et al. [37] and Evans et al. [38], who reported statistically significant differences in lateral core endurance in favor of men, the current study revealed no statistically significant differences in core endurance data across the different core muscle groups. To the best of our knowledge, no other study has reported statistically significant higher core power values for men in flexion or right and left lateral flexion than for women. Further research is needed to determine the differences in maximal core strength, core endurance, and core power in terms of sex as well as other aspects, such as sports or age, due to the limited evidence currently available [29,70]. A larger empirical data base could facilitate the adaptation of sport-specific training and prevention programs to individual needs.

### Limitations and Future Directions

The core strength diagnostics used in this study are less representative due to its lack of inclusion of the seated and standing test positions in flexion, extension, and right and left lateral flexion during the maximal core strength, core power, and core endurance measurements. The results found in this study depend on specific test conditions with explicit body angles in the lying position during individual exercises (flexion, extension, and lateral flexion right and left). Therefore, different body angles and positions can lead to different results in core strength measurement. Tomčić et al. [42] showed differentiated components of maximal isometric core strength for seated and standing positions. They assumed that the transmission of mechanical force within the myofascial chain differed between the seated and standing positions. Consequently, the involvement of the hip and lower body muscles could affect the core strength in the sagittal and frontal planes. Further research is necessary to investigate the differences in core strength components in lying, seated, and standing positions. Additionally, the test type, namely isometric measurement, did not fully reflect the demands of core strength in sporting movements overall. In particular, the linear direction of movement in the measurement of the maximal strength and power of the lateral core muscles rarely occurs in sporting movements. Therefore, dynamic measurements of maximal core strength and core power may be more helpful if sport-specific measurements are to be conducted. Since many sports, such as tennis (e.g., tennis serve), baseball (e.g., pitching), and handball (e.g., jump shot), involve increased rotational movements, it would be useful to measure the rotational dynamic force of the lateral core muscles [16,20,27]. Nevertheless, the objective of this study was to identify the potential components of core strength, with an isometric measurement in a laboratory setting being preferred owing to enhanced control through a higher standardization of the test conditions than in dynamic measurement.

## 5. Conclusions

The present study indicates three principal components of the core strength construct on the basis of a PCA of twelve variables that were measured isometrically in holding time, MVC, and pRFD measurements in the same exercises and in a lying position. The maximal core strength, core power, and core strength predominantly represent the core strength construct and are not related to each other. These results suggest that sport practitioners think more holistically and measure all three components of core strength isometrically and dynamically to train these components according to individual needs to potentially improve sports performance and/or reduce the risk of injury.

## Figures and Tables

**Table 1:** Descriptive statistics of holding time, MVC, and pRFD measurements in different exercises.

Variable	Flexion		Extension		Lateral Flexion Right		Lateral Flexion Left	
	*M*	*SD*	*M*	*SD*	*M*	*SD*	*M*	*SD*
Holding time (s)	220.52	137.98	160.76	48.92	65.19	28.36	72.88	28.68
pRFD (N/s)	1869.29	943.53	1570.98	819.25	743.69	354.94	745.08	450.52
Timepoint pRFD (ms)	1569.44	297.95	1548.62	293.29	1402.33	366.41	1475.30	455.11
Time to pRFD (ms)	211.37	37.60	205.71	43.82	223.57	63.57	213.30	64.00
MVC (N)	296.70	101.76	314.73	85.30	150.73	70.49	164.68	72.85
Timepoint MVC (ms)	3310.36	739.23	3682.03	917.84	3097.13	757.26	3396.68	776.78
Time to MVC (ms)	1959.49	672.02	2343.52	832.81	1934.42	657.20	2129.48	706.13
Start point (ms)	1350.87	286.06	1338.51	296.03	1162.71	372.92	1267.21	469.29
Start force (N)	56.66	23.87	55.65	25.66	37.84	24.96	44.83	34.70

Note: *M*: mean value; MVC: maximal isometric voluntary contraction; pRFD: peak rate of force development; *SD*: standard deviation.

**Table 2:** Within-session reliability of MVC and pRFD variables.

Variable	*ICC*	95% CI	*CV* (%)	*SEM*
**MVC**				
Flexion	0.99	(0.97, 0.99)	5.51	12.46
Extension	0.96	(0.93, 0.98)	8.38	17.06
Lateral flexion right	0.98	(0.96, 0.99)	11.67	10.71
Lateral flexion left	0.96	(0.93, 0.98)	11.62	14.20
**pRFD**				
Flexion	0.88	(0.79, 0.94)	29.14	386.83
Extension	0.84	(0.73, 0.91)	29.24	408.40
Lateral flexion right	0.83	(0.69, 0.91)	23.94	236.42
Lateral flexion left	0.88	(0.80, 0.94)	24.41	201.36

Note: CI: confidence interval; *CV*: coefficient of variation; *ICC*: intraclass correlation coefficient; MVC: maximal isometric voluntary contraction; pRFD: peak rate of force development; *SEM*: standard error of measurement.

## Data Availability

The original contributions presented in this study are included in the article/Supplementary Materials. Further inquiries can be directed to the corresponding author.

## Supplementary Materials

The following supporting information can be downloaded at https://www.mdpi.com/article/10.3390/jfmk9040249/s1.
