# Supplementary material for: The Multi-Component Structure of Core Strength
Source: J Funct Morphol Kinesiol. 2024 Nov 28;9(4):249. doi: 10.3390/jfmk9040249 (PMC11678708; doi:10.3390/jfmk9040249)
Supplement: Supplementary file 1 [file jfmk-09-00249-s001.zip › Supplementary Materials.pdf]

Supplementary Materials

Sample size calculation

The sample size was determined using the formula by Giraudeau and Mary [1]:

$$n = \frac{8z_{1-\alpha/2}^{2}\,(1-ICC)^{2}\,[1+(m-1)\,ICC]^{2}}{m(m-1)\,w^{2}} \quad (z_{1-\alpha/2} = 1.96;\ ICC = 0.9;\ m = 3;\ w = 0.1) \qquad (1)$$

Note. $z_{1-\alpha/2}$ = quantile of standard normal distribution, *m* = number of measurements, *ICC* = intra-class correlation coefficient, *w* = total width of the 100(1-α) % confidence interval

Table S1. Correlations of holding time, MVC, and pRFD variables

Variable	Holding time				MVC				pRFD			
	Flexion	Extension	Lateral flexion right	Lateral flexion left	Flexion	Extension	Lateral flexion right	Lateral flexion left	Flexion	Extension	Lateral flexion right	Lateral flexion left
**Holding time**												
Flexion	-											
Extension	0.542	-										
Lateral flexion right	0.420	0.585	-									
Lateral flexion left	0.391	0.589	0.881	-								
**MVC**												
Flexion	0.210	-0.052	0.261	0.328	-							
Extension	0.277	0.190	0.338	0.356	0.565	-						
Lateral flexion right	0.272	0.200	0.612	0.610	0.689	0.619	-					
Lateral flexion left	0.141	0.166	0.467	0.523	0.693	0.608	0.835	-				
**pRFD**												
Flexion	0.138	0.110	0.292	0.383	0.581	0.218	0.471	0.386	-			
Extension	-0.001	0.034	0.164	0.316	0.275	0.363	0.303	0.255	0.520	-		
Lateral flexion right	0.165	0.063	0.352	0.455	0.513	0.379	0.453	0.377	0.498	0.701	-	
Lateral flexion left	-0.149	-0.126	0.149	0.297	0.392	0.247	0.380	0.413	0.593	0.536	0.643	-

Note. Correlation coefficients (Pearson's *r*) between variables are shown. MVC: maximal isometric voluntary contraction, pRFD: peak rate of force development

Table S2. Differences in holding time, MVC, and pRFD variables by sex

Variable	female (*n* =20)		male (*n* = 22)						
	M	*SD*	*M*	*SD*	*t*(40)	*p*	*g*	95% CI	
**Holding time**									
Flexion	232.20	118.54	209.91	155.60	0.52	0.607	0.16	-0.44	0.75
Extension	171.85	52.97	150.68	43.70	1.42	0.164	0.43	-0.17	1.03
Lateral flexion right	60.40	27.73	69.55	28.85	-1.05	0.302	-0.32	-0.91	0.28
Lateral flexion left	67.40	20.76	77.86	34.07	-1.19	0.242	-0.36	-0.96	0.24
**MVC**									
Flexion	216.15	64.37	369.93	68.34	-7.49	<0.001	-2.27	-3.04	-1.49
Extension	278.73	58.40	347.47	93.60	-2.82	0.007	-0.86	-1.47	-0.23
Lateral flexion right	108.75	53.30	188.91	62.66	-4.44	<0.001	-1.37	-2.00	-0.68
Lateral flexion left	120.67	44.12	204.70	71.26	-4.54	<0.001	-1.38	-2.04	-0.70
**pRFD**									
Flexion	1465.50	732.30	2236.37	984.01	-2.87	0.007	-0.87	-1.49	-0.24
Extension	1449.23	606.21	1681.65	975.17	-0.92	0.365	-0.28	-0.87	0.32
Lateral flexion right	566.40	301.31	904.86	326.60	-3.48	0.001	-1.06	-1.69	-0.41
Lateral flexion left	548.54	351.18	923.76	463.09	-2.94	0.005	-0.89	-1.51	-0.26

Note. CI: confidence interval, *M*: mean value, MVC: maximal isometric voluntary contraction, pRFD: peak rate of force development, *SD*: standard deviation
